# miR-15a-5p regulates myocardial fibrosis in atrial fibrillation by targeting Smad7

**DOI:** 10.7717/peerj.12686

**Published:** 2021-12-20

**Authors:** Dan He, Zhong-bao Ruan, Gui-xian Song, Ge-cai Chen, Fei Wang, Mei-xiang Wang, Mao-kun Yuan, Li Zhu

**Affiliations:** 1 Department of Cardiology, Jiangsu Taizhou People’s Hospital, Taizhou, China; 2 Dalian Medical University Graduate School of Medicine, dalian, China; 3 Department of Cardiothoracic Surgery, Jiangsu Taizhou People’s Hospital, Taizhou, China

**Keywords:** MiR-15a-5p, Myocardial fibrosis, Smad7, TGF-β1, Atrial fibrillation

## Abstract

**Background:**

At present, there is no effective treatment for myocardial fibrosis in atrial fibrillation (AF). It is reported that miR-15a-5p is abnormally expressed in AF patients but its specific role remains unclear. This study aims to investigate the effect of miR-15a-5p in myocardial fibrosis.

**Methods:**

Left atrial appendage (LAA) tissues were collected from AF and non-AF patients. In lipopolysaccharide (LPS) stimulated H9C2 cells, miR-15a-5p mimic, inhibitor, pcDNA3.1-Smad7 and small interfering RNA-Smad7 (siRNA-Smad7) were respectively transfected to up-regulate or down-regulate the intracellular expression levels of miR-15a-5p and Smad7. Quantitative real-time polymerase chain reaction (qRT-PCR) and western blot (WB) were used to determine the expression levels of miR-15a-5p, Smad7, transforming growth factor β1 (TGF-β1) and collagen I. Cell counting kit-8 (CCK-8) and ethylene deoxyuridine (EdU) were used to determine cell viability and proliferation capacity, respectively. Dual-luciferase was used to detect whether miR-15a-5p interacted with Smad7, hydroxyproline (HYP) and Hematoxylin-Eosin (HE) staining were used to detect tissue fibrosis.

**Results:**

The expression levels of miR-15a-5p, TGF-β1 and collagen I were up-regulated, while Smad7 was down-regulated in AF tissues and LPS-stimulated cells. MiR-15a-5p mimic can inhibit the expression of Smad7, and the dual-luciferase experiment confirmed their interaction. MiR-15a-5p inhibitor or pcDNA3.1-Smad7 can inhibit LPS-induced fibrosis and cell proliferation, while siRNA-Smad7 can reverse the changes caused by miR-15a-5p inhibitor.

**Conclusion:**

We combined clinical studies with LPS-stimulated H9C2 cell model to validate the role of miR-15a-5p in the regulation of Smad7 and fibrosis. Taken together, the miR-15a-5p/Smad7 pathway might be a potential target for AF therapy.

## Introduction

Atrial fibrillation (AF), one of the most common arrhythmias in clinical practice, is still one of the main causes of stroke, heart failure, sudden death and cardiovascular disease globally, with a high disability and mortality rate. With an aging population, the incidence and prevalence of AF continue to rise, and the social burden is increasing (Lippi, Sanchis-Gomar & Cervellin, 2021). Studies have shown that the major pathological basis of AF is atrial remodeling, including atrial electrical remodeling, structural remodeling, and nerve remodeling. Myocardial fibrosis plays a key role in the occurrence and development of atrial structural remodeling (Wijesurendra & Casadei, 2019). It is now believed that myocardial fibrosis and the development of AF have a mutually reinforcing effect, but the exact pathogenesis is not fully understood.

A large number of studies have confirmed that transforming growth factor β1 (TGF-β1) is the most closely related factor to tissue fibrosis, mainly through the TGF-β1/Smad classical signaling pathway (Meng, Nikolic-Paterson & Lan, 2016). The Smad protein family is the key regulator of TGF-β1, and up-regulated of Smad7 can reduce the pulmonary fibrosis caused by bleomycin (Wang et al., 2016). In contrast, down-regulated of Smad7 expression is involved in fibrosis induced by AF in both human and animal models (He et al., 2016; Li et al., 2019a).

MiRNA is conserved 18–25 nucleotide noncoding RNA that modulates gene expression by base-pairing with the 3′ untranslated regions of mRNAs and induces mRNA degradation or translational inhibition of the target. MiRNA is involved in a variety of biological processes, including cell proliferation, differentiation, apoptosis and inflammation (Shen et al., 2020). Abnormal expression of miRNA is usually related to pathological diseases (Li et al., 2016). Circulating miR-15a-5p has been found to be a biomarker for diffuse myocardial fibrosis in patients with hypertrophic cardiomyopathy (Fang et al., 2015). Li et al. (2019b) previously used bioinformatics to predict that miRNA-15a might be directly related to the Smad7 gene and might be involved in the pathogenesis of AF. However, the specific mechanisms of miR-15a-5p and Smad7 in AF have not been investigated yet.

In this study, we measured the differential expression levels of miR-15a-5p and Smad7 from LAA tissues in patients with AF. Lipopolysaccharide (LPS) is an important part of gram-negative bacteria, which can induce sepsis and related heart damage (Li et al., 2020). Circulating LPS also induces cardiac fibrosis and alters left ventricular structure and function (Asgharzadeh et al., 2018). LPS can induce the levels of fibrosis-related factors FGF-2, uPA, MMP-2 and MMP-9 through the ERK1/2 pathway in H9C2 cells (Han et al., 2017). Therefore, we further investigated the role of miR-15a-5p and Smad7 in LPS-stimulated H9C2 cells and explored its molecular basis to provide new therapeutic avenues for AF.

## Materials and methods

### Human myocardial tissue collection

The left atrial appendage (LAA) of 20 patients who underwent valve replacement at the Cardiothoracic Surgery at Department of Taizhou People’s Hospital were collected from June 2020 to June 2021. This study was conducted in accordance with the principles of the Declaration of Helsinki and was approved by the Research Ethics Committee of Taizhou People’s Hospital (approval number: KY202010901). The LAA tissue samples were divided into the control group and AF group (*n* = 10, each) according to the ECG results and medical histories. All the LAA tissue samples were collected with the consent of the patients and their family members, and the informed consents were signed. After the valve replacement cardiopulmonary bypass was established, about 200 mg of LAA tissue of the patients was taken, the blood was washed away with physiological saline, and the fat tissue was removed with tissue scissors and divided into pre-prepared cryotubes, and the samples were numbered and stored in a refrigerator at −80 °C for later use. The appropriate amount of tissues were fixed in formalin and embedded.

### Quantitative real-time polymerase chain reaction (qRT-PCR)

Total RNAs from tissues and cells were isolated using an RNA extraction kit. According to the manufacturer’s protocol, reverse transcription was performed using the AMV first-strand cDNA synthesis kit. In order to determine the expression level of miRNA, we synthesized cDNA with special stem-loop primers specific to the target miRNA. Expression levels of miRNA and mRNA were measured according to the manufacturer’s instructions for the 2X SG Fast qPCR master mix. U6 and glyceraldehyde 3-phosphate dehydrogenase (GAPDH) were used to standardize the expression levels of miRNA and mRNA, respectively. The difference of expression levels between the samples was calculated using the 2-ΔΔCt method. All qRT-PCR kits and primers were purchased from Sangon Biotech, China. The primer sequences used for qRT-PCR are shown in Table 1.

**Table 1:** List of primers used for qRT -PCR.

Gene	Forward primer	Reverse primer
TGF-β	AGCAACAATTCCTGGCGATACCTC	TCAACCACTGCCGCACAACTC
collagen I	TTCTCCTGGCAAAGACGGAC	CTCAAGGTCACGGTCACGAA
Smad7	GCAGAAATCCAAGCACCACCAAAC	CAGCCGCACACTCACACTCAC
GAPDH	ACGTGTCAGTGGTGGACCTG	GTGTAGCCCAGGATGCCCTT
miRNA-15a-5p	CGCGTAGCAGCACATAATGG	AGTGCAGGGTCCGAGGTATT
U6	AGAGAAGATTAGCATGGCCCCTG	ATCCAGTGCAGGGTCCGAGG
miRNA-15a-5p RT	GTCGTATCCAGTGCAGGGTCCGAGGTATTCGCACTGGATACGACCACAAA	
U6 RT	GTCGTATCCAGTGCAGGGTCCGAGGTATTCGCACTGGATACGACAAAATA	

### Western blot analysis (WB)

RIPA lysate (Beyotime, China) and protease inhibitor (Biosharp, China) were used to extract proteins from tissues and cells, and the protein concentration was determined by the BCA protein concentration determination kit (Beyotime, China). The proteins were isolated by sodium dodecyl sulfate-polyacrylamide gel electrophoresis (SDS-PAGE) after deanaturation, and then the proteins were transferred to a polyvinylidene fluoride (PVDF) membranes (Millipore, China). PVDF membranes were blocked with 5% skimmed milk at room temperature with shaking for 2 h, then incubated with the primary antibody at 4 °C overnight, and the secondary antibody at room temperature for 1.5 h. Clarity TM Western ECL Substrate Kit (Bio-Rad, Hercules, CA, USA) was used to detect proteins. Primary antibodies include anti-GAPDH (1:5, 000, ab181602; Abcam, Britain), anti-TGF-β1 (1:1, 000, ab179695; Abcam, UK), anti-Smad7 (1:1, 000, ab90086; Abcam, Britain), anti-collagen I (1:1, 000, ab260043; Abcam, Britain), and secondary antibody, goat anti-rabbit IgG H&L (HRP) (Sangon Biotech, China). GAPDH was used to normalize protein expression levels.

### Determination of hydroxyproline (HYP) level

According to the manufacturer’s instructions of the HYP detection kit (Solarbio, USA), the HYP in the LAA tissues was extracted and was measured the optical density (OD value) of the sample in a microplate reader (Thermo Scientific, Waltham, MA, USA) at a wavelength of 560 nm. The HYP level was calculated according to the proline content calculation formula.

### Hematoxylin-Eosin (HE) staining

The LAA tissues were collected and fixed in formaldehyde for 24 h, then embedded in paraffin. The sections were used for the following steps: dewaxing, water coating, staining, dehydration, dewaxing, and finally sealed with neutral gum. The tissue sections (nucleus stained with hematoxylin and cytoplasm stained with eosin) were observed under a light microscope.

### Cell culture and transfection

H9C2 cells were purchased from the Shanghai Institute of Biochemistry and Cell Biology, Chinese Academy of Sciences. They were cultured in a DMEM medium (Gibco, USA) containing 10% fetal bovine serum (FBS; Hyclone, USA) and 1% double antibiotics (Gibco, USA), moist air (5% CO_2_) at 37 °C. Before transfection, H9C2 cells were seeded in a 6-well plate, antibiotic-free medium was added, and the cells were 50–60% confluent after 12-24 h. The cell culture medium was changed to a serum-free medium to prepare for transfection. The transfection process was performed according to the instructions of Lipofectamine^®^ 3000 (L3000015; Invitrogen, USA). Cells were stimulated with 1 µ g/ml LPS, and 24 h later, miR-15a-5p inhibitor-NC (negative control), miR-15a-5p inhibitor or pcDNA3.1, pcDNA3.1-Smad7 or siRNA-Smad7 were transfected into the cells, and the specific groups were: A: Control, LPS, LPS + miR-15a-5p inhibitor-NC, LPS + miR-15a-5p inhibitor; B: Control, LPS, LPS +pcDNA3.1, LPS +pcDNA3.1-Smad7; C: Control, LPS, LPS + miR-15a-5p inhibitor, LPS + miR-15a-5p inhibitor + siRNA-Smad7, 48 h after transfection, the cells were collected for WB, qRT-PCR, CCK8 and EdU analysis.

### Cell viability determination

Cell viability was detected by using a highly water-soluble tetrazolium salt cell counting kit-8 (CCK-8; Cellorlab, China). The cell suspension (100 µl/well) was seeded into a 96-well plate, and 10 µl of CCK-8 solution was added to each well after pre-incubation (37 °C, 24 h) in an incubator. Subsequently, the plate was placed in an incubator for 0-72 h; OD values at a wavelength of 450 nm were measured at 0, 24, 48, and 72 h using a microplate reader (Thermo Scientific). According to the formula cell survival rate = (experimental group OD value/control group OD value) ×100%.

### 5-Ethynyl-2′-deoxyuridine (EdU) assay

The proliferation of H9C2 cells was determined using the Click-iT EdU imaging kit (Thermo Scientific) according to the manufacturer’s protocol. Cells were treated for 24 h, incubated with 10 µM EdU for 1 h, fixed in 4% paraformaldehyde for 20 min, followed by permeabilization and EdU staining. Nuclei were re-stained by incubation with 0.5ug/ml DAPI for 15 min protected from light. Imaging was carried out under a fluorescence microscope.

### Dual-luciferase reporter assay

The predicted binding sequence was identified and downloaded from TargetScan (http://www.targetscan.org). The recombinant pmirGLO plasmid containing the binding sequence was constructed by Meft Biotech Co, Ltd (Jiangsu, China). To confirm whether Smad7 is an effective target of miRNA-15a-5p, H9C2 cells were used for dual-luciferase reporter gene detection. This fragment contains the wild-type (WT) or mutant (MUT) Smad7 3′UTR region and is cloned into the pmirGLO dual-luciferase miRNA target expression vector. Then the pmirGLO vector miR-15a-5p mimic and Lipofectamine3000 (L3000015; Invitrogen, USA) were co-transfected into H9C2 cells. After 24 h of co-transfection, the luciferase activity was detected by the dual-luciferase reporter gene detection system (Promega, USA). Firefly luciferase was standardized by Renilla luciferase. Total RNA and protein were collected and extracted 48 h after cell transfection.

### Statistical analysis

The data were analyzed by using GraphPad Prism 8.0.1 software. The data were expressed as mean ± standard error of the mean (SEM). The significant difference between the two groups was analyzed by Student’s t-test; The difference among more than two groups was analyzed by a one-way analysis of variance (ANOVA) followed by post-hoc tests. *P* < 0.05 was considered to be statistically significant.

## Results

### The role of miR-15a-5p and Smad7 in myocardial fibrosis in patients with AF

**Figure 1:**
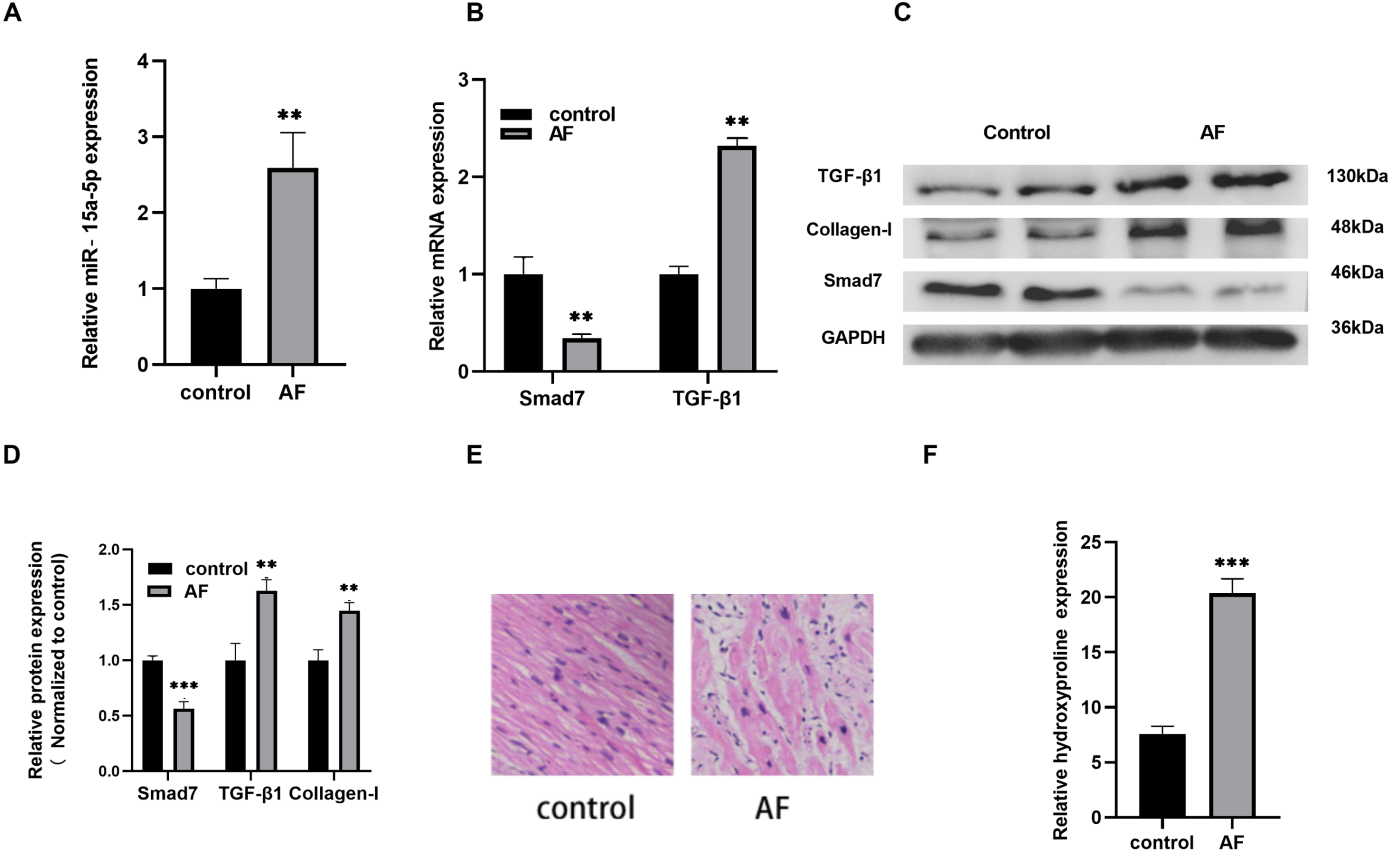
The expression level of miR-15a-5p and Smad7 in LAA tissues. (A, B) Expression levels of miR-15a-5p, Smad7 and TGF-β1 were detected by qRT-PCR; (C, D) The expression levels of three related proteins (TGF-β1, collagen I, Smad7) were measured by western blot. (E) HE staining was used to show typical microscopy of myocardial tissue fibrosis image; (F) Hydroxyproline was used to determine the degree of tissue fibrosis. ** *P* < 0.01 *vs.* control group; *** *P* < 0.001 *vs.* control group.

Compared with the control group, the expression level of miR-15a-5p in AF was increased significantly (Fig. 1A), and the expression level of TGF-β1, a key regulator of fibrosis, was up-regulated while Smad7 was down-regulated (Figs. 1B, 1C, 1D). The protein expression of the fibrosis marker collagen I in AF was increased (Figs. 1C, 1D). At the same time, HE staining revealed that the cardiomyocyte hypertrophy and atrophy of the AF group were intertwined, the cell morphologies were irregular, the arrangements were disordered, and the interstitial myocardial cells were significantly increased (Fig. 1E). In addition, myocardial fibrosis was evaluated by measuring the HYP content in LAA tissues. The HYP content in AF was higher than that in the control group (Fig. 1F). Together, the above results indicate that the up-regulated of miR-15a-5p and the down-regulated of Smad7 might play a role in myocardial fibrosis in patients with AF.

### Identification of LPS-stimulated H9C2 cells and miR-15a-5p expression

**Figure 2:**
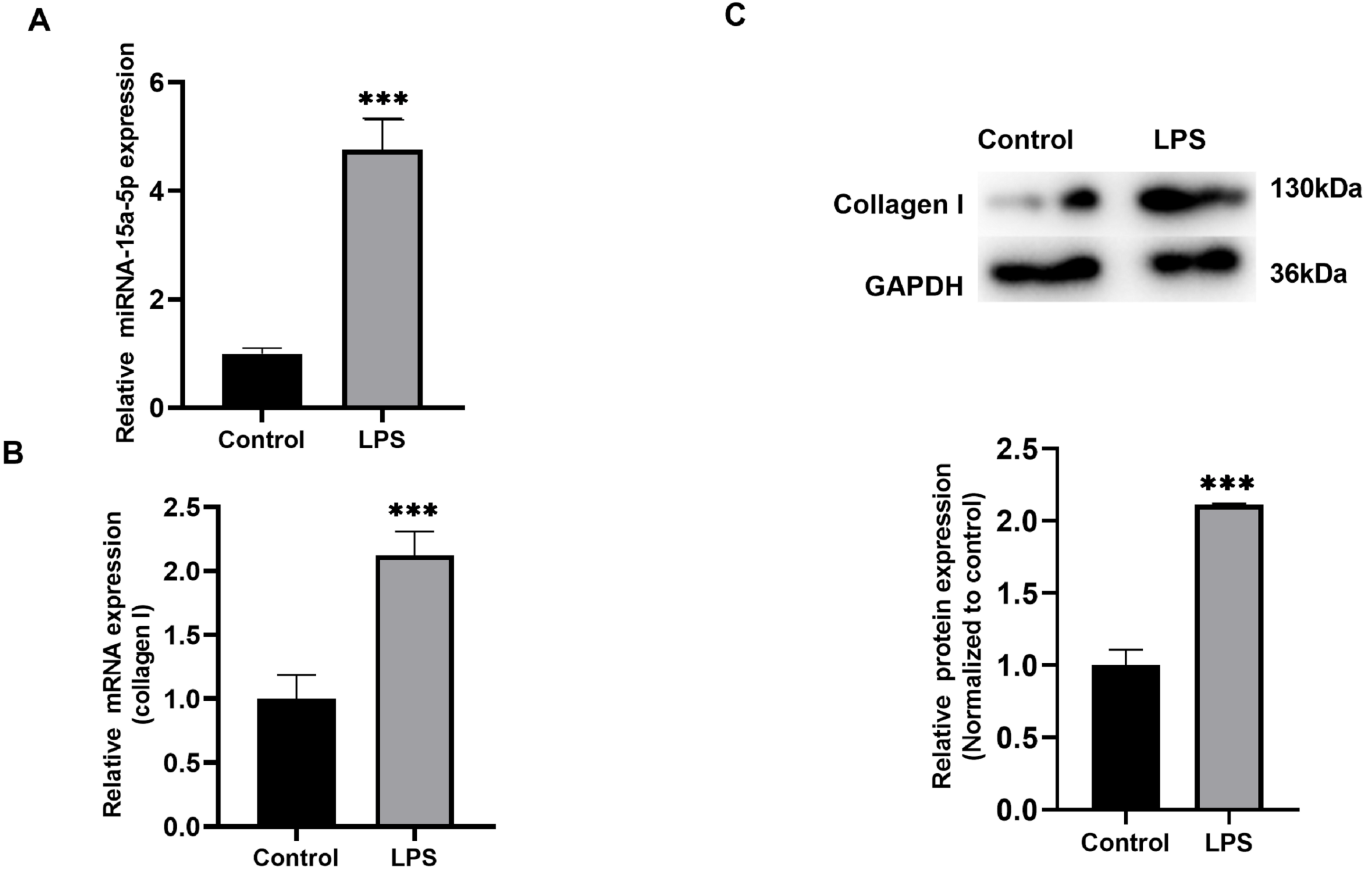
The expression level of miR-15a-5p in the LPS-stimulated H9C2 fibrosis cell model. (A) Expression level of miR-15a-5p in LPS-stimulated H9C2 cells was measured by the qRT-PCR; (B, C) qRT-PCR and western blot assay were utilized to detect the expression level of collagen I in LPS-stimulated H9C2 cells. *** *P* < 0.001 *vs.* control group.

H9C2 cells cultured in vitro were stimulated with LPS (1 µg/ml), qRT-PCR results showed that the expression level of miR-15a-5p was significantly higher than that of the control group (Fig. 2A), and collagen I was also differentially expressed in the stimulation group (Fig. 2B). WB analysis verified that the expression level of collagen I was significantly higher than that of the control group (Fig. 2C).

### Fibrosis were regulated by Smad7 in LPS-stimulated H9C2 cells

**Figure 3:**
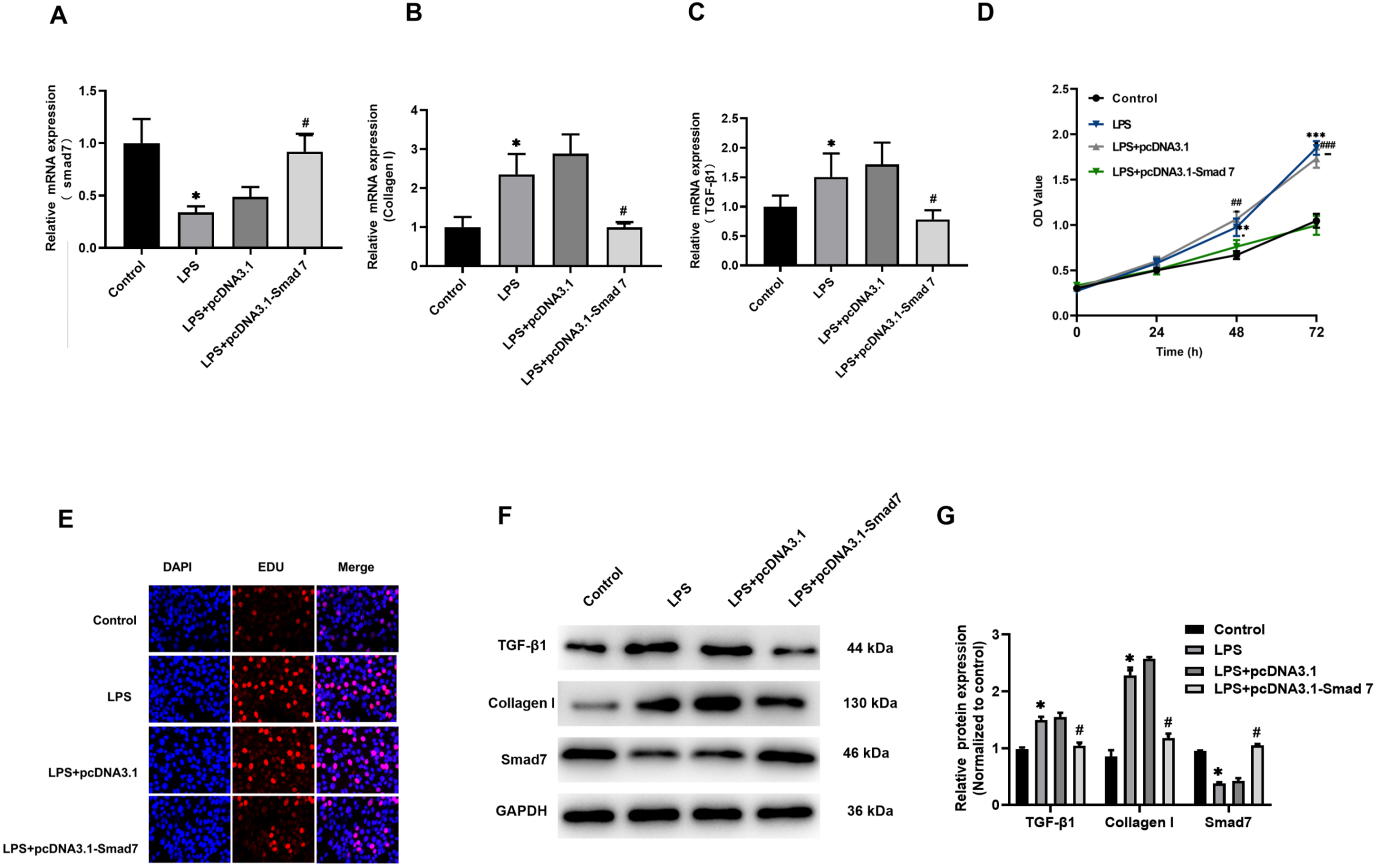
Fibrosis was regulated by Smad7. (A, B, C) pcDNA1.3 targeting Smad7 or negative control (NC) was transfected into LPS-induced H9C2 cells. Expression levels of Smad7, TGF-β1 and collagen I were analyzed by qRT-PCR in each group; (D, E) CCK8 and EdU were used to show the cell viability and proliferation capacity; (E, G) Western blot analysis was used to measure the expression levels of Smad7, TGF-β1, and collagen I in different groups. * *P* < 0.05 LPS group *vs.* control group; # *P* < 0.05 pcDNA3.1 group *vs.* pcDNA3.1-Smad7 group.

According to our results reported in tissues, Smad7 expression was down-regulated and collagen I expression was up-regulated in patients with AF. In order to study the role of Smad7 in myocardial fibrosis, we transfected pcDNA3.1 or pcDNA3.1-Smad7 into LPS-stimulated H9C2 cells, the expression level of Smad7 in the pcDNA3.1-Smad7 group was higher than that in the pcDNA3.1 group (Fig. 3A). The results of qRT-PCR and WB showed that the expression level of collagen I in LPS-stimulated H9C2 cells was significantly inhibited by Smad7 (Figs. 3B, 3F, 3G). In addition, Smad7 had been shown to reduce the expression level of TGF-β1 (Figs. 3B, 3F, 3G). CCK8 and EdU results demonstrated that the cell viability and proliferation capacity were lower in pcDNA3.1-Smad7 group (Figs. 3D, 3E). In summary, these results indicate that Smad7 might play an important role in LPS-stimulated H9C2 cells.

### MiR-15a-5p directly targets Smad7 in H9C2 cells

**Figure 4:**
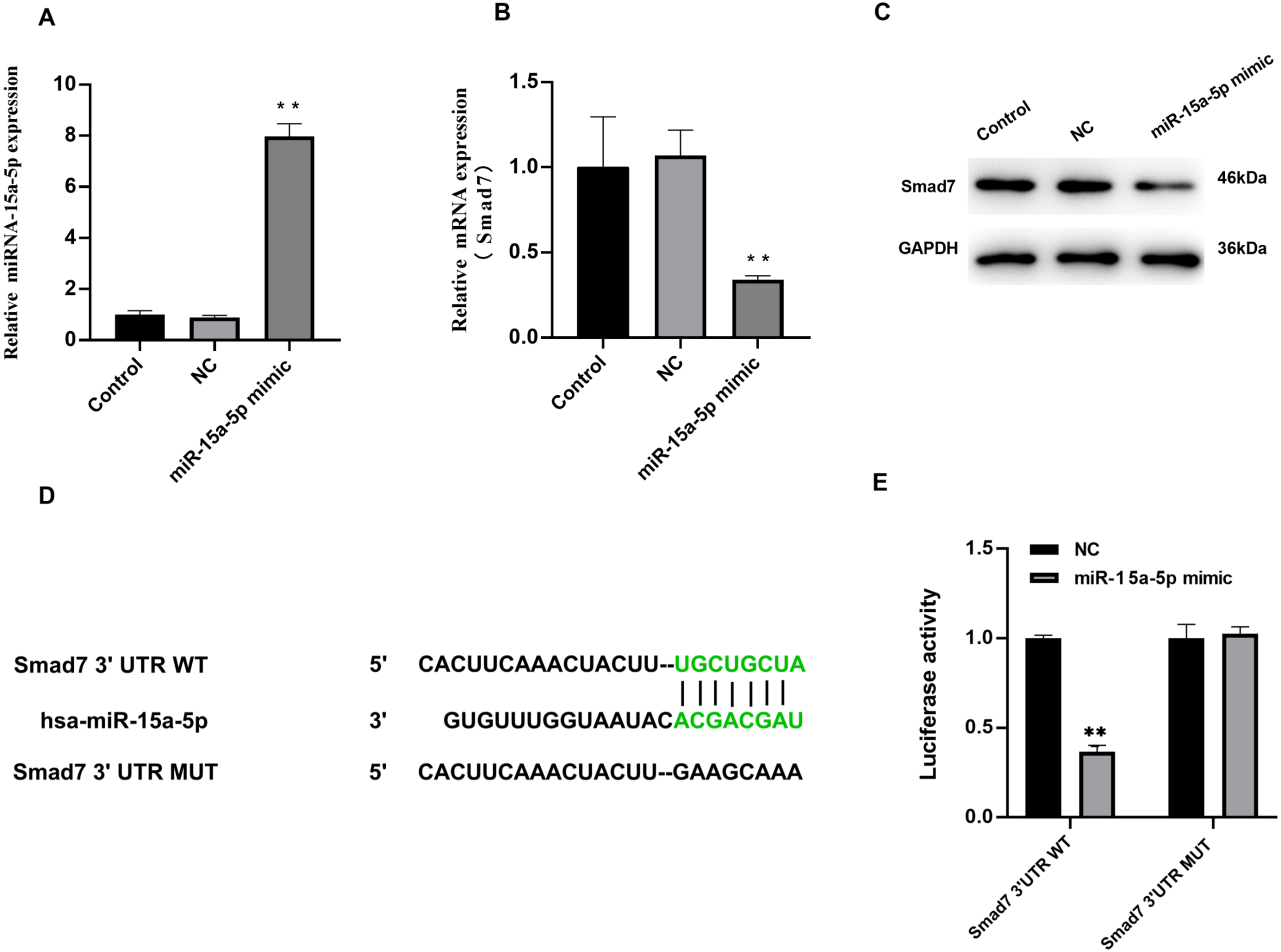
Smad7 was a direct target of miR-15a-5p. (A) miR-15a-5p mimic or negative control (NC) was transfected into H9C2 cells and the transfection efficiency was measured by the qRT-PCR; (B, C) Smad7 expression was detected by the qRT-PCR and western blot in different groups; (D) The putative binding site between miR-15a-5p and Smad7 mRNA was predicted by target scanning; (E) H9C2 cells were cotransfected with a luciferase reporter and miR-15a-5p mimic. Data were presented as the relative ratio of firefly to Renilla luciferase activity. ** *P* < 0.01 *vs.* NC group. NC, Negative control.

We investigated the effect of miR-15a-5p overexpression on Smad7. In H9C2 cells transfected with miR-15a-5p mimics, qRT-PCR results showed significantly enhanced expression of miR-15a-5p (Fig. 4A), while Smad7 expression was significantly down-regulated in both qRT-PCR and WB results (Figs. 4B, 4C). We used TargetScan to predict that the 3′UTR of Smad7 had a target site for miR-15a-5p (Fig. 4D). To validate this combined effect, we constructed a dual fluorescent reporter system. WT or MUT sequences of the predicted target binding sequence in Smad7 mRNA were co-transfected with miR-15a-5p mimic and luciferase into H9C2 cells. Enzyme activity assays showed that overexpression of miR-15a-5p significantly reduced the enzyme activity of the Smad7 3′UTR WT group (Fig. 4E). These results indicate that miR-15a-5p might regulate the expression of the Smad7.

### Down-regulated of miR - 15a -5p in LPS-stimulated H9C2 cells inhibits collagen I and promotes Smad7 expression

**Figure 5:**
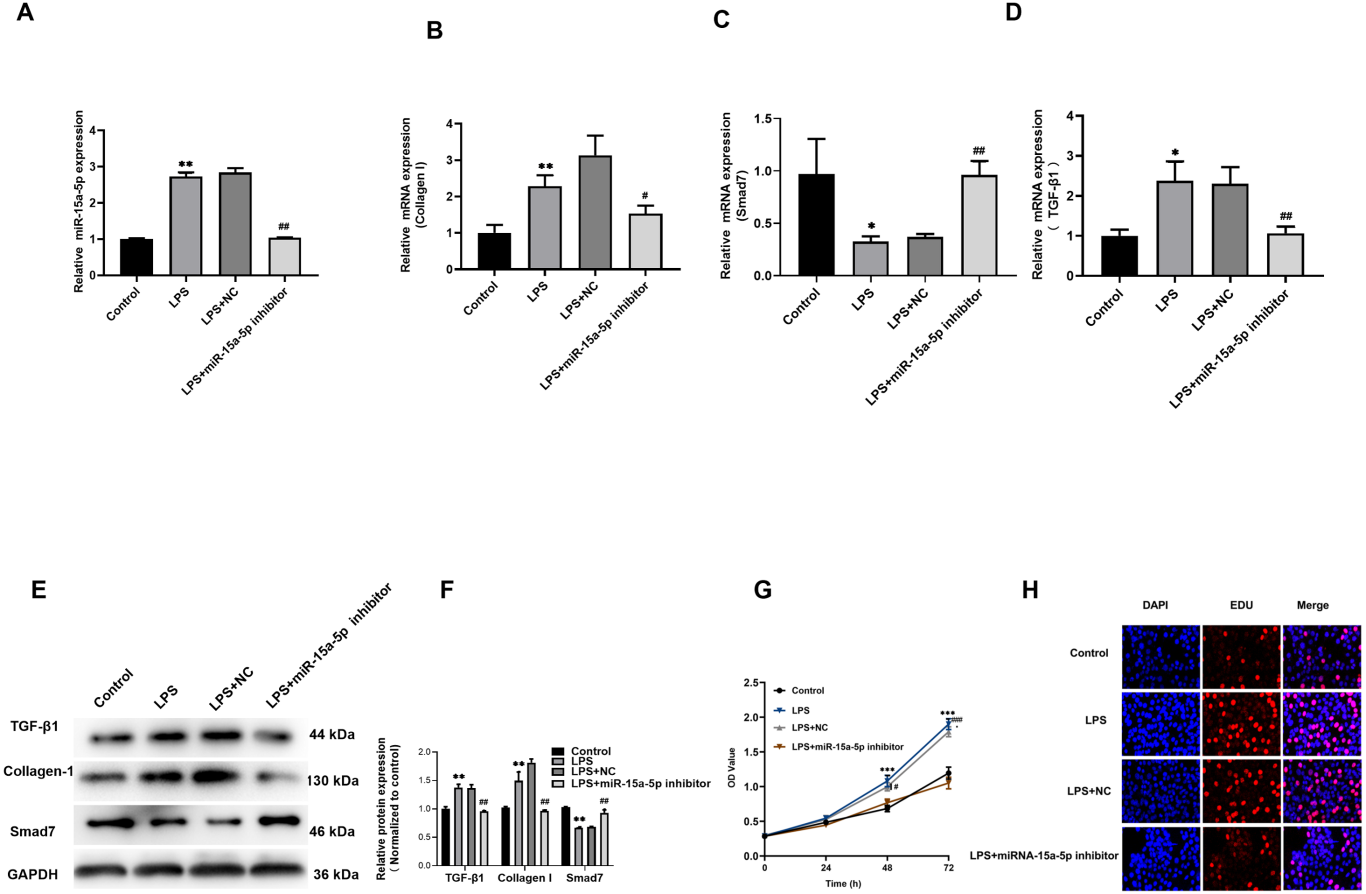
Down-regulated of miR-15a-5p can inhibit collagen I and promote Smad7 expression. (A, B, C, D) Expression of miR-15a-5p, TGF-β1, collagen I, and Smad7 in different groups were evaluated by the qRT-PCR; (E, F) The expression levels of three related proteins (TGF-β1, collagen I, Smad7) were measured in four groups by western blot after transfection. (G, H) CCK8 and EdU were utilized to detect the effect of transfection on the cell viability and proliferation capacity; * *p* < 0.05 LPS group *vs.* control; ** *p* < 0.01 LPS group *vs.* Control; # *p* < 0.05 miR-15a-5p inhibitor group *vs.* NC group; ## miR-15a-5p inhibitor group *vs.* NC group. NC: Negative control.

The miR-15a-5p inhibitor or negative control was transfected in LPS-stimulated H9C2 cells. The results showed that the upregulation of TGF-β1, collagen I and the down-regulated of Smad7 were reversed by miR-15a-5p inhibitor (Figs. 5A–5F). CCK8 and EdU results showed that cell viability and proliferation capacity were significantly reduced in the LPS+ miR-15a-5p inhibitor group compared to the LPS+ NC group (Figs. 5G, 5H). These results suggest that miR-15a-5p might play an important role in the regulation of Smad7 and collagen I in LPS-stimulated H9C2 cells.

### TGF-β1/collagen I might be the downstream signaling pathway of miR-15a-5p/Smad7

**Figure 6:**
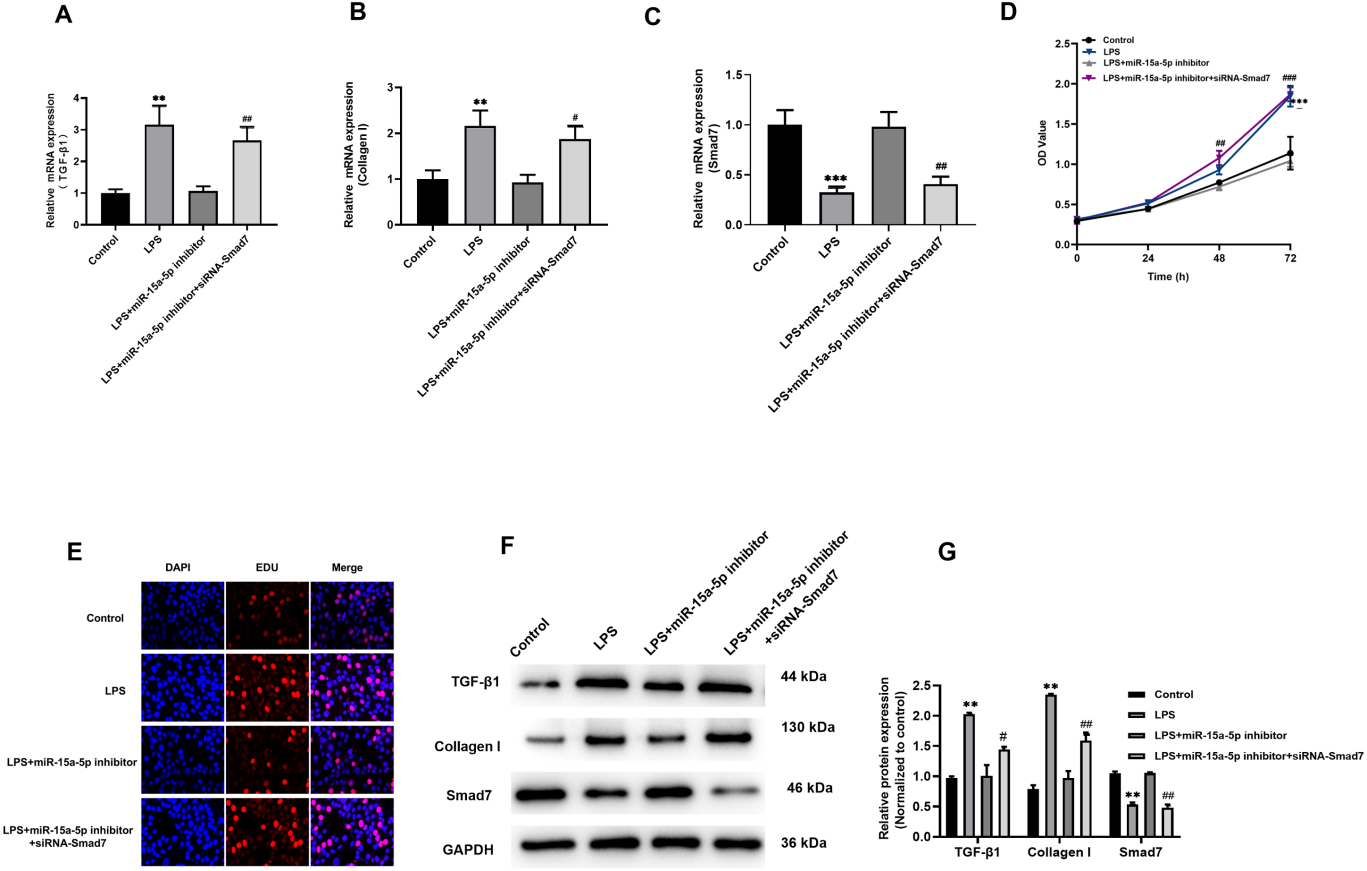
TGF-β1/collagen I may be the downstream signaling pathway of miR-15a-5p/Smad7. (A, B, C) SiRNA-Smad7 was transfected to miR-15a-5p inhibitor group. TGF-β1, collagen I and Smad7 in different groups were tested by qRT-PCR; (D, E) CCK8 at different times (0, 24, 48, 72 h) and EdU showed the effect of transfection on the cell viability and proliferation capacity; (F, G) Expression of TGF-β1, collagen I, Smad7 were measured by western blot. ** *p* < 0.01 LPS group *vs.* control; ^***^ *p* < 0.001 LPS group *vs.* Control; # *p* < 0.05 miR-15a-5p inhibitor group *vs.* siRNA-Smad7 group; ## *p* < 0.01 miR-15a-5p inhibitor group *vs.* siRNA-Smad7 group.

The above results indicate that LPS induces up-regulated of TGF-β1 and collagen I expression in H9C2 cells, while Smad7 down-regulated. However, pcDNA3.1-Smad7 or miR-15a-5p inhibitor can inhibit the expression levels of TGF-β1, collagen I and Smad7 in LPS-stimulated H9C2 cells. In addition, we further investigated and found that siRNA-Smad7 could suppress the inhibitory effect of the miR-15a-5p inhibitor on the gene and protein expression levels of TGF-β1, collagen I and Smad7 (Figs. 6A–6C, 6F and 6G). CCK8 and EdU results demonstrated that the cell viability and proliferation capacity were dramatically higher in the siRNA-Smad7 group than in the miR-15a-5p inhibitor group (Figs. 6D, 6E). These results indicate that TGF-β1/collagen I might be an important signal transduction pathway downstream of miR-15a-5p/Smad7.

## Discussion

AF is an important factor of myocardial fibrosis, and fibrosis plays an important role in the occurrence and development of AF. Studies have shown that LPS can induce cardiomyocyte fibrosis (Chen et al., 2019). Although acute subclinical levels of LPS in the circulation are usually tolerated, the persistence of even subclinical levels of LPS can trigger cardiac fibrosis (Lew et al., 2013). In the current study, we observed that compared with the control group, miR-15a-5p was up-regulated in the LAA tissue of patients with AF while Smad7 was down-regulated. At the same time, the content of fibrosis index HYP and the expression level of collagen I increased in the AF group. The in vitro cell model stimulated by LPS showed that miR-15a-5p and Smad7 expression was consistent with that in tissues, leading to increased expression of fibrotic marker in cardiomyocytes. In cultured H9C2 cells, we found that miR-15a-5p can regulate the expression of Smad7 by directly binding to its mRNA 3′-UTR. Down-regulated of miR-15a-5p or up-regulated of Smad7 in LPS-stimulated H9C2 cells reversed the expression of fibrotic marker. Further experiments showed that TGF-β1/collagen I was activated by LPS and regulated by miR-15a-5p/Smad7. These results suggest that miR-15a-5p might play a role in AF fibrosis by regulating Smad7 mRNA.

Studies have shown that many members of the miR-15 family are up-regulated soon after birth and could help to inhibit some cell cycle genes and cell cycle arrest (Porrello et al., 2011). Subsequent studies proved that inhibiting the miR-15 family from as early as birth to adulthood can increase the proliferation of cardiomyocytes in the adult heart and improve the left ventricular systolic function after myocardial infarction in adults (Porrello et al., 2013). As for fibrosis, some studies have demonstrated the fibrotic effect of miRNA-15a. In the heart of diabetic patients characterized by progressive myocardial fibrosis, the expression of miRNA-15a in biopsy tissue was significantly down-regulated, while TGF-β1 and connective tissue growth factor (CTGF) were significantly up-regulated before fibrosis. In addition, the therapeutic repair of miRNA-15a in cardiomyocytes reduces the activation of TGF-β1 and CTGF, reduceing the differentiation of cardiac fibroblasts in diabetic patients (Yong et al., 2016). This study was the first to determine the role of miR-15a-5p in myocardial fibrosis in AF. Compared with the control group, the expression of miR-15a-5p in patients with AF was significantly increased, and we further studied its mechanism of action. In vitro experiments, miRNA inhibitor was transfected to restore the expression of miR-15a-5p in LPS-stimulated H9C2 cells, inhibited the proliferation and differentiation induced by LPS, and reduced the fibrosis of cardiomyocytes. These results indicate that miR-15a-5p is an important pro-fibrotic factor and might become a new target for AF treatment.

Smad7 is an inhibitory protein in the Smad family. Studies have shown that in other pathological conditions, such as myocardial fibrosis in AF, which is attributed to the neurohumoral activation effect, the level of Smad7 is down-regulated (He et al., 2016). Research by Meng et al. (2020) in a mouse hypertensive heart disease model showed that increasing Smad7 is expected to block Smad3-mediated myocardial fibrosis. In the current study, the expression levels of Smad7 in tissues of AF patients and LPS-stimulated H9C2 cells were consistent with previous findings and were associated with fibrosis. In addition, the TGF-β1/collagen I signaling pathway in LPS-stimulated H9C2 cells can be blocked by siRNA-Smad7. Smad7 was an upstream regulator of fibrosis, and increasing Smad7 expression can reverse the cell proliferation and the expression of cellular fibrotic marker.

Studies in other diseases have shown that miR-15a can bind and regulate Smad7 mRNA and participate in fibrosis (Liu et al., 2015). However, there is no research to determine whether miR-15a-5p and Smad7 are related to the pathogenesis of myocardial fibrosis in AF. Previous bioinformatics methods have shown that miRNA-15a and Smad7 may be involved in the pathogenesis of AF (Li et al., 2019b). TGF-β1 is involved in many biological processes and is the most classical regulator of fibrosis. Smad7 is involved in the regulation of TGF-β1, which is one of the important regulatory mechanisms of myocardial fibrosis in AF. Therefore, we chose to study whether miR-15a-5p and Smad7 were involved in myocardial fibrosis by affecting the TGF-β1/collagen I pathway. Our results showed that miR-15a-5p expression was up-regulated, while Smad7 expression was down-regulated in patients with AF. At the same time, we found that overexpression of miR-15a-5p in H9C2 cells can lead to a decrease in Smad7 expression. Our study confirmed that miR-15a-5p overexpression could negatively regulate the expression of Smad7. We further used target scanning to reveal that miR-15a-5p could interact with Smad7, with the results showing that 8 nucleotides in the 3′-UTR of Smad7 mRNA matched the sequence of miR-15a-5p. To confirm this prediction, we performed a dual-luciferase reporter system test. The results showed that the presence of miR-15a-5p mimic significantly reduced the enzyme activity, indicating that the predicted sequence had a binding effect. The above results validated that Smad7 was a target gene for miR-15a-5p.

The above study showed that miR-15a-5p down-regulated or Smad7 up-regulated reversed LPS-stimulated proliferation and fibrosis in H9C2 cells. In addition, siRNA-Smad7 can reverse miR-15a-5p inhibitor down-regulated of TGF-β1 and collagen I and upregulation of Smad7. miRNA-15a-5p/Smad7 might be an effective target for the treatment of fibrosis in AF, while TGF-β1/collagen I may be its downstream signaling pathway. However, this study has some limitations: The tissue sample size was relatively small, and there may be bias in the results, and a larger, multicenter study is warranted in the future. In addition, although we validated our findings in tissues and H9C2 cells, we still need to confirm our results in cardiac fibroblasts and animal models at a later stage.

In summary, this study found that differential expression of miRNA-15a-5p and Smad7 in patients with AF was associated with fibrosis. We confirmed the expression of miR-15a-5p and Smad7 in a fibrotic cell model constructed from H9C2 cells, and we also uncovered the direct targeting relationship between miR-15a-5p and the 3′-UTR of Smad7 mRNA. Furthermore, we further reveal that TGF-β1/collagen I might be a downstream signaling pathway to miRNA-15a-5p/Smad7. In light of our findings, we provide a potential avenue for the effective treatment for AF.

## Supplemental Information

10.7717/peerj.12686/supp-1 Supplemental Information 1 Fig. 1-1 raw data

10.7717/peerj.12686/supp-2 Supplemental Information 2 Fig. 1-2 raw data

10.7717/peerj.12686/supp-3 Supplemental Information 3 Fig. 2 raw data

10.7717/peerj.12686/supp-4 Supplemental Information 4 Fig. 3 raw data

10.7717/peerj.12686/supp-5 Supplemental Information 5 Fig. 4 raw data

10.7717/peerj.12686/supp-6 Supplemental Information 6 Fig. 5 raw data

10.7717/peerj.12686/supp-7 Supplemental Information 7 Fig. 6 raw data

## Additional Information and Declarations

### Data Availability

The following information was supplied regarding data availability: The raw measurements are available in the Supplemental File.

### Clinical Trial Registration

The following information was supplied regarding Clinical Trial registration: KY202010901
